# Improving antibiotic prescribing for pediatric acute respiratory tract infections: A cluster randomized trial to evaluate individual versus clinic feedback

**DOI:** 10.1017/ash.2021.212

**Published:** 2021-11-03

**Authors:** Herbert W. Clegg, Stephen J. Ezzo, Kelly B. Flett, William E. Anderson

**Affiliations:** 1 Novant Health and Novant Health Medical Group, Charlotte, North Carolina; 2 Center for Outcomes Research and Evaluation, Atrium Health, Charlotte, North Carolina

**Keywords:** antimicrobial, antibiotic, stewardship, audit, feedback, peer, pediatric, acute, respiratory, tract, infections, cluster, randomized, trial

## Abstract

**Objective:**

To assess the effect of individual compared to clinic-level feedback on guideline-concordant care for 3 acute respiratory tract infections (ARTIs) among family medicine clinicians caring for pediatric patients.

**Design:**

Cluster randomized controlled trial with a 22-month baseline, 26-month intervention period, and 12-month postintervention period.

**Setting and participants:**

In total, 26 family medicine practices (39 clinics) caring for pediatric patients in Virginia, North Carolina, and South Carolina were selected based upon performance on guideline-concordance for 3 ARTIs, stratified by practice size. These were randomly allocated to a control group (17 clinics in 13 practices) or to an intervention group (22 clinics in 13 practices).

**Interventions:**

All clinicians received an education session and baseline then monthly clinic-level rates for guideline-concordant antibiotic prescribing for ARTIs: upper respiratory tract infection (URI), acute bacterial sinusitis (ABS), and acute otitis media (AOM). For the intervention group only, individual clinician performance was provided.

**Results:**

Both intervention and control groups demonstrated improvement from baseline, but the intervention group had significantly greater improvement compared with the control group: URI (odds ratio [OR], 1.62; 95% confidence interval [CI], 1.37–1.92; *P* < 0.01); ABS (OR, 1.45; 95% CI, 1.11–1.88; *P* < 0.01); and AOM (OR, 1.59; 95% CI, 1.24–2.03; *P* < 0.01). The intervention group also showed significantly greater reduction in broad-spectrum antibiotic prescribing percentage (BSAP%): odds ratio 0.80, 95% CI 0.74-0.87, *P* < 0.01. During the postintervention year, gains were maintained in the intervention group for each ARTI and for URI and AOM in the control group.

**Conclusions:**

Monthly individual peer feedback is superior to clinic-level only feedback in family medicine clinics for 3 pediatric ARTIs and for BSAP% reduction.

**Trial registration:**

ClinicalTrials.gov identifier: NCT04588376, Improving Antibiotic Prescribing for Pediatric Respiratory Infection by Family Physicians with Peer Comparison.

Ambulatory settings account for ∼60% of antibiotic expenditures,^1^ and it has been estimated that ∼30% of antibiotic use in these settings is unnecessary.^2^ In ambulatory settings, audit and feedback has been shown to decrease inappropriate antibiotic prescribing and was recently recommended among the core elements of ambulatory stewardship.^3–12^ Behavioral science research shows individual data provision for quality improvement may be more effective than group data.^13^ A recent before-and-after, quasi-experimental quality improvement initiative in 22 pediatric clinics in our healthcare system showed marked improvement in guideline-concordant care for 3 acute respiratory tract infections (ARTIs) when individual peer comparison data were provided monthly over time.^7^ However, provision of these individual feedback reports is time- and resource-consuming and may not provide improvement over group feedback. In fact, we found no trials of ambulatory antimicrobial prescribing feedback comparing individual clinician performance with feedback on group performance.

In this context, we designed a cluster randomized trial to directly compare the effect of individual peer comparison feedback to group feedback on guideline-concordant care for 3 pediatric ARTIs among family medicine clinicians. Family medicine clinics were the focus for 2 reasons. First, previous studies show significant variability in guideline-concordant care for pediatric ARTIs between pediatricians and non-pediatricians.^14–16^ Secondly, there are limited antimicrobial stewardship data for pediatric patients cared for by family medicine clinicians. We hypothesized that individual feedback would be superior to group feedback in improving appropriate antibiotic prescribing.

## Methods

### Trial design

In August 2015, retrospective review of data from January 1, 2014, to June 30, 2015, was conducted by the team. We then set eligibility criteria for a parallel group, cluster-randomized, controlled trial as follows: (1) family medicine practices within the healthcare system (n = 85); (2) upper respiratory tract infection or common cold measure (URI) of <83% (mean score in 2013 Healthcare Effectiveness Data Information Set in patients aged 3 months to 18 years,^17^ n = 34); (3) ≥20 pediatric illness encounters with a diagnosis of URI in the previous 6 months (n = 28); and (4) consent by the lead clinician by e-mail for practice participation (n = 26). A practice consisted of at least 1 cluster or clinic site, and several practices in the system had multiple sites.

### Participants

The 85 family medicine practices (some of which had multiple sites or clinics) in North Carolina, South Carolina, and Virginia, affiliated with 13 hospitals in the Novant Health system, included 341 physicians and 143 advanced practice providers. Novant Health uses Epic software (EpicCare,Verona, WI) for their electronic health records. The analytics and informatics group develops data sets from which quality and safety measurement data are abstracted. Our ambulatory antimicrobial stewardship team, developed in 2013, consisted of 2 quality specialists, a pharmacist, and 4 physicians (a pediatrician and 3 infectious disease specialists).

### Interventions

All participating practices received the following:

1. A 1-hour, in-person, educational session, in September–October 2015, with the lead clinician (and occasionally other clinicians at the clinic), clinic administrator, and stewardship team physicians describing the project and clinical guidelines for URI, acute bacterial sinusitis (ABS), and acute otitis media (AOM).^18–21^
2. A tip sheet detailing how to improve scores (including appropriate codes and documentation strategies) (Supplementary Digital Content 1 online).
3. An after-visit summary for clinicians to give to patients and parents discussing antibiotic use and side effects (Supplementary Digital Content 2 online).
4. Clinic-level baseline performance data for all pediatric and family medicine clinics for the 3 ARTIs. Following the educational session, baseline and then monthly data were provided by e-mail to the lead clinician and clinic administrator. They were asked to forward via e-mail or to hand deliver the data to clinicians at the site and to share the data at monthly clinic meetings. Compliance with information flow to individual clinicians was measured only informally through multiple follow-up discussions in-person or via email with each clinic. The lead clinicians in the control group received clinic-level performance data only (Supplementary Digital Content 3 online), and lead clinicians in the intervention group additionally received clinician-specific data with individual comparisons limited to the specific site (Supplementary Digital Content 3 and 4 online). Thus, all practices could view performance data for all pediatric and family medicine practices by practice name at the clinic level. Intervention practices could also view individual scores by clinician name for their clinic sites but not others. For the very few clinicians who practiced in >1 site, performance data were provided for each site. Clinical decision support was not used.
5. Discussion about a new clinical pathway for acute bacterial sinusitis with a request for adoption and implementation.

Interventions were applied and measured at the cluster (ie, clinic) level and not at the patient level.

### Data collection

Baseline performance data were collected retrospectively from January 1, 2014, through October 31, 2015. The intervention period was November 1, 2015, through December 31, 2017. The postintervention period was January 1 through December 31, 2018, during which time only clinic-level data were provided monthly to all 39 clinics. Visit-level data included *International Classification of Disease, Ninth Revision* (ICD-9) codes and, starting in October 2015, ICD-10 codes (see Supplementary Digital Content 5 online) associated with a patient’s illness encounter and listed as a visit diagnosis. Illness encounters were defined as evaluation and management visits for new patients (with codes 99201–99205) and for established patients (with codes 99212–99215).

### Outcomes

Using guidelines from the American Academy of Pediatrics^18–20^ and the Infectious Diseases Society of America,^21^ customized clinical quality measures for the 3 ARTIs were developed and validated by selective, manual electronic chart review of the electronic record.

In brief, ARTIs were defined as follows. URI was defined as the percentage of children aged 3 months to 18 years diagnosed with URI who were not dispensed an antibiotic prescription on or within 3 days after the illness encounter. ABS was defined as the percentage of children aged 1–18 years diagnosed with ABS who were dispensed a first-line antibiotic (ie, amoxicillin or amoxicillin-clavulanate). AOM was defined as the percentage of children aged 6 months to 12 years diagnosed with AOM who were dispensed the first-line antibiotic (amoxicillin). Illness encounters were excluded if an antibiotic was prescribed within 30 days prior (if URI) or within 60 days prior (if ABS or AOM) to the illness encounter (for complete measure definitions, see Supplementary Digital Content 5 online).

The proportions of illness encounters receiving appropriate care for each ARTI over the baseline, intervention, and postintervention periods, respectively, were measured for each site as the number of illness encounters with appropriate care divided by the total number of illness encounters involving the ARTI. The primary outcomes were the relative changes in the proportion of illness encounters receiving appropriate care between the baseline and intervention periods for the 3 ARTIs. The same assessments between the intervention and postintervention periods were secondary outcomes. A target of ≥90% for URI was set using the HEDIS 2013 90th percentile^17^ and, at 80% for ABS and AOM, consistent with targets suggested by an outpatient antibiotic use target-setting workgroup.^22^

Other secondary outcomes were the baseline-to-intervention period change and intervention-to-postintervention period change in broad-spectrum antibiotic prescribing percentage (BSAP%), determined monthly by enumerating all antibiotics given for pediatric illness encounters, stratifying by narrow and broad spectrum (see Table 3 for definitions of each) and dividing by the total number of antibiotics prescribed for any condition, not limited to the 3 ARTIs. For this calculation, patients were excluded if their record showed an allergy to an antibiotic listed as narrow or broad spectrum and/or one of the listed antibiotics had been given in the prior 60 days.

To determine whether code shifting occurred or total antibiotic utilization changed after the intervention began, we recorded the mean number of encounters per clinic for the 3 ARTIs and the total of all pediatric illness encounters and antibiotics prescribed for all pediatric patients seen for illness in the baseline and intervention periods in both groups.

### Randomization

Although outcomes were assessed at the clinic level, we randomized at the practice level to avoid intrapractice contamination of the intervention. The 26 participating practices were stratified by size: very large (2 practices, each with 5–10 clinic sites), large (4 practices with ≥9 clinicians), small (10 practices with 6–8 clinicians), and very small (10 practices with <6 clinicians). All but the very large practices were at a single site.

**Fig. 1.**
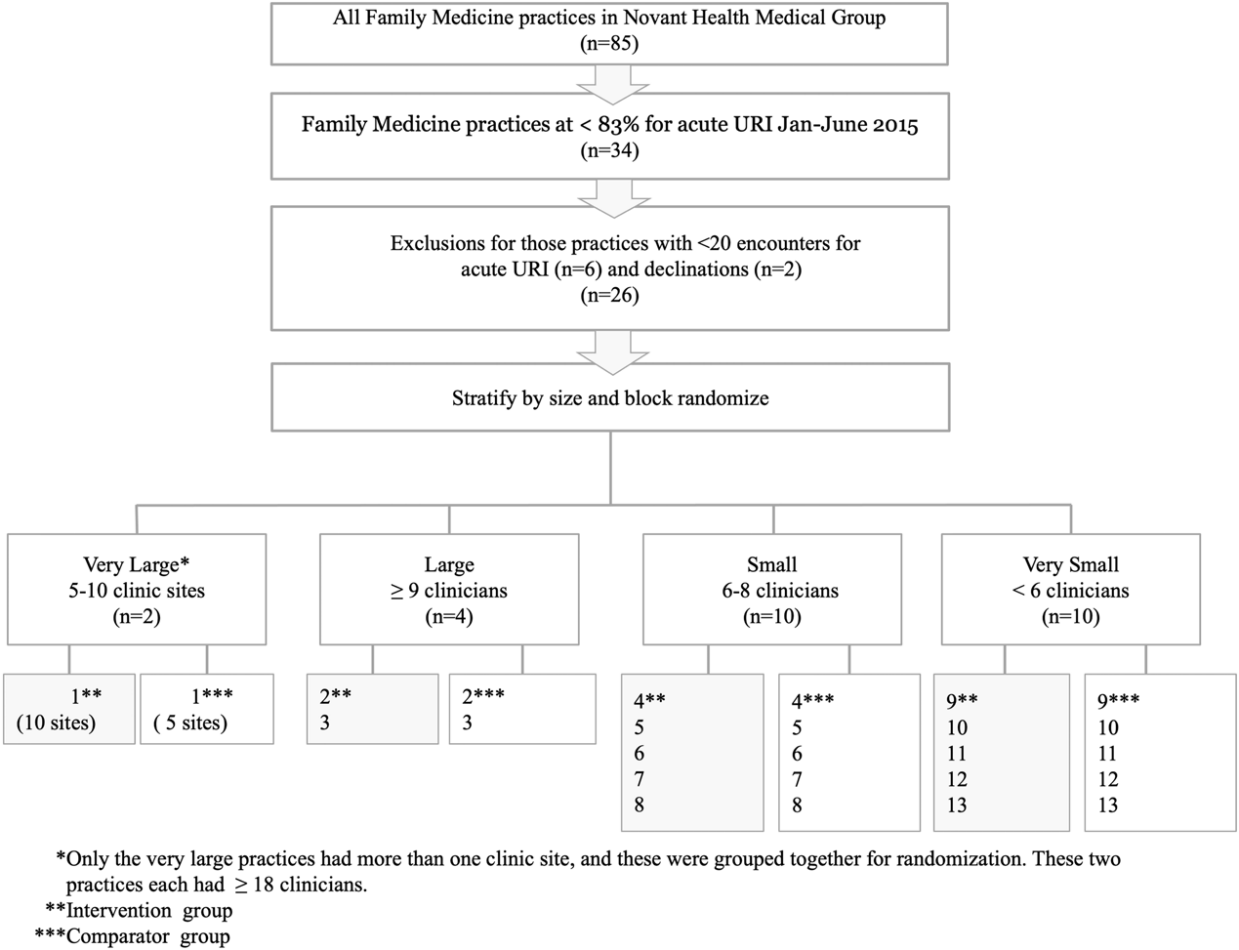
Flow diagram for clinic selection process.

Using PROC SURVEYSELECT in SAS software,^23^ half of the practices in each stratum were randomly selected to be in the intervention group, and the remaining practices were assigned to the control group (see Fig. 1).

Practices were unaware of randomization allocation until educational visits occurred. Practices could not be masked after allocation, and all continuously participated for the duration of the trial. Evaluators were unmasked after randomization.

### Statistical methods

Power was calculated at the cluster (ie, clinic) level and was based on the primary outcomes, the change in appropriate care between the baseline and intervention periods for the 3 ARTIs. Following randomization of the available practices, the numbers of clusters were determined to be 22 (22 clinics in 13 practices) for the intervention group and 17 (17 clinics in 13 practices) for the control group. Based on retrospective data from the baseline period, the average cluster sizes (ie, the average numbers of encounters per clinic over 24 months or the average length of the baseline and intervention periods) for URI, ABS, and AOM, respectively, were estimated to be 229.1, 79.2, and 140.9 for the intervention group and 230.2, 71.5, and 145.7 for the control group. These data provided >85% power to detect a study group difference in mean baseline-to-intervention period change of 0.4 standard deviations, corresponding to a medium effect size^24^ with 2-sided α = 0.05 for intracluster correlations ranging between 0.01 and 0.15. The power analysis was performed using PASS 15 software.^25^

The clinic was the unit of analysis for describing changes in clinical decision making, whereas the illness encounter was the unit of observation. Clinic-level baseline variables are summarized for the intervention and control groups in Table 1.

**Table 1.** Baseline Characteristics of Practice Sites Allocated to Intervention and Control Groups

Characteristics of Practice Sites	Intervention Group (n=22), Mean (SD) per Clinic	Control Group (n=17), Mean (SD) per Clinic
Illness encounters per month	164.5 (99.2)	159.1 (131.5)
Antibiotic prescriptions per month	41.8 (27.5)	40.2 (31.7)
Antibiotic prescribing clinicians per month	5.6 (3.0)	5.9 (3.9)
Illness encounters per clinician per month	32.0 (21.3)	24.9 (7.9)
**Illness encounters for each ARTI per month**		
URI	9.5 (6.8)	9.6 (7.6)
ABS	3.3 (3.0)	3.0 (2.7)
AOM	5.9 (3.5)	6.1 (5.7)

Note. ARTI, acute respiratory tract infection; URI, upper respiratory infection; ABS, acute bacterial sinusitis; AOM, acute otitis media.

All other analyses were performed using SAS software,^23^ and graphics were created using R software.^26^ To assess the influence of the intervention on clinical decision making, a generalized linear mixed model (GLMM) was analyzed for each outcome using PROC GLIMMIX in SAS software. The response variable in the models was of the form y/n, where y is the number of appropriate treatment occurrences and n is the total number of relevant encounters. The response distribution was specified as binomial with a logit link function. The independent variables were the study group (intervention and control), measurement period (baseline, intervention, and after intervention), and a study-group-by-period interaction term. The models included random-intercept terms at both the clinic and practice levels to account for relatedness of clinical decisions made in the same clinic or practice. The group-by-period interaction term was the estimate of interest used to compare the baseline period-to-intervention period (or intervention-to-postintervention period) relative change in appropriate prescribing between the intervention and control groups. Adjusted odds ratios and corresponding 95% confidence intervals were used to quantify the group difference in prescribing change. Analysis of the secondary outcome, BSAP%, was conducted using the same approach.

To account for multiple testing, the Holm step-down procedure^27^ was used to adjust *P* values, where the overall α level was prespecified at 0.05 and the family of inferences included the tests of group-by-period interaction for all the aforementioned models.

For each outcome variable, the intra-cluster correlation (ICC) was calculated using the level-2 (ie, random intercept for clinic) variance from the GLMM and a level-1 variance component assumed to be π^2^/3 = 3.29 for a logistic random intercept model. The ICC is a quantitative measure of within-cluster correlation, defined as the proportion of the total variance in the outcome attributed to the variance between clusters.^28^

### Ethical considerations

The protocol was reviewed and approved by the Institutional Review Board of Novant Health Presbyterian Medical Center, Charlotte, North Carolina.

## Results

Figure 1 details the flow of participants. The intervention group (13 practices and 22 clinics) had 124 clinicians during the baseline period and 134 clinicians during the intervention period. For the control group (13 practices and 17 clinics), there were 101 clinicians during the baseline period and 103 for the intervention period. During the 4-year period, there were 173,131 pediatric illness encounters in the intervention group and 126,125 pediatric illness encounters in the control group. All clinics participated throughout all 3 periods studied.

**Fig. 2.**
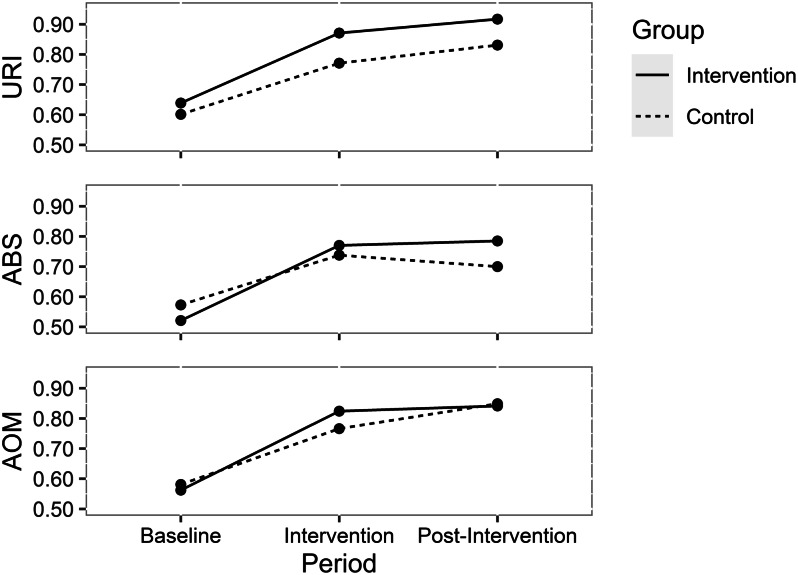
Proportion of illness encounters with appropriate prescribing for the intervention and control groups during the 3 periods studied.

Both groups were comparable at baseline with respect to all variables examined (Table 1). Both groups demonstrated improvement during the intervention period compared to baseline for each ARTI as shown graphically (Fig. 2). The intervention group improved performance for URI by 23.2% (absolute percentage change), ABS by 24.9%, and AOM by 26.2% (mean improvement for all 3 of 24.8%). The control group improved for URI by 17.0%, ABS improved by16.5%, and AOM improved by 18.5%, with mean improvement overall of 17.3% (Table 2).

**Table 2.** Comparison of the Percentage of Patients With Appropriate Treatment for ARTI in Intervention and Control Groups for 3 Study Periods

Variable	Intervention Group		Control Group			
	Proportion With Appropriate Treatment^a^	%	Proportion With Appropriate Treatment	%	Adjusted Odds Ratio (95% CI)^b^	*P* Value^b^
URI						
Baseline period	2,950/4,619	63.9	2,157/3,587	60.1		
Intervention period	6,319/7,252	87.1	3,476/4,508	77.1		
Postintervention period	489/533	91.7	461/555	83.1		
Change from baseline to intervention period		23.2		17.0	1.62 (1.37–1.92)	<.01
Change from intervention period to postintervention period		4.6		6.0	0.98 (0.60–1.60)	.93
ABS						
Baseline period	832/1,597	52.1	638/1,113	57.3		
Intervention period	1,817/2,359	77.0	1,421/1,925	73.8		
Postintervention period	833/1,061	78.5	692/988	70.0		
Change from baseline to intervention period		24.9		16.5	1.45 (1.11–1.88)	<.01
Change from intervention period to postintervention period		1.5		−3.8	1.42 (1.05–1.92)	.02
AOM						
Baseline period	1,599/2,843	56.2	1,319/2,272	58.1		
Intervention period	1,702/2,066	82.4	1,268/1,655	76.6		
Postintervention period	900/1,090	82.6	615/724	84.9		
Change from baseline to intervention period		26.2		18.5	1.59 (1.24–2.03)	<.01
Change from intervention period to postintervention period		0.2		8.3	0.63 (0.44–0.92)	.02

Note. ARTI, acute respiratory tract infection; URI, upper respiratory infection; ABS, acute bacterial sinusitis; AOM, acute otitis media.

a Proportions are totals for patients with appropriate treatment for the specific ARTI/specific pediatric illness encounters.

b Adjusted odds ratios and *P* values are generated from the generalized linear mixed model (GLMM) and compare the intervention and control groups on the change from baseline to intervention period, and on the change from the intervention period to the postintervention period. *P* values are adjusted for multiple testing.

**Table 3.** Comparison of Broad-Spectrum Antibiotic^a^ Prescribing Percentage in Intervention and Control Groups for 3 Study Periods

Variable	Intervention Group		Control Group		Adjusted Odds Ratio (95% CI)^b^	*P* Value^b^
	Proportion of Total Antibiotics Prescribed That Were Broad Spectrum	%	Proportion of Total Antibiotics Prescribed That Were Broad Spectrum	%		
Baseline period	8,855/17,527	50.5	6,060/13,521	44.8		
Intervention period	7,646/18,963	40.3	5,878/14,880	39.5		
Postintervention period	2,257/6,562	34.4	1,987/5,530	35.9		
Change from baseline to intervention period		−10.2		−5.3	0.80 (0.74–0.87)	<.01
Change from intervention period to postintervention period		−5.9		−3.6	0.87 (0.78–0.97)	.02

a Broad-spectrum antibiotics include amoxicillin-clavulanate, azithromycin, cefaclor, cefdinir, cefixime, cefpodoxime, ceprozil, ceftriaxone, cefuroxime, ciprofloxacin, clarithromycin, clindamycin, erythromycin, levofloxacin, linezolid, loracarbef, and moxifloxacin.

b Adjusted odds ratios and *P* values are generated from the generalized linear mixed model (GLMM) and compare the intervention and control groups on the change from baseline to intervention period, and on the change from the intervention period to the postintervention period. *P* values are adjusted for multiple testing.

However, the intervention group had significantly greater improvement compared with the control group. For URI, the odds ratio (OR) was 1.62 (95% confidence interval [CI], 1.37–1.92; *P* < .01). For ABS, the OR was 1.45 (95% CI, 1.11–1.88; *P* < .01), and for AOM, the OR was 1.59 (95% CI, 1.24–2.03; *P* < .01) (Table 2).

When individual peer data were withdrawn for the intervention group for January–December 2018, gains in performance were sustained and scores continued to increase for all 3 measures comparing the intervention period and the postintervention period: URI increased from 87.1% to 91.7%; ABS increased from 77.0% to 78.5%; and AOM increased from 82.4% to 82.6%. For the control group, performance improved for URI from 77.1% to 83.1% and for AOM performance improved from 76.8% to 84.9% (Table 2). Neither group met the goal of ≥80% for ABS. The control group’s performance declined in the postintervention period for ABS from 73.8% to 70.0% (Table 2).

For the secondary outcome, reduction in BSAP%, the baseline mean for the intervention group was 50.5% and for the control group it was 44.8%. The 2 groups reached nearly identical means for the intervention period (40.3% for the intervention group and 39.5% for the control group) and continued to decrease in the postintervention period. However, reduction in BSAP%, comparing baseline-to-intervention periods, was significantly greater for the intervention group (OR, 0.80; 95% CI, 0.74–0.87; *P*< .01). Further declines were noted in the postintervention period to 34.4% and 35.9%, respectively, for the intervention and control groups (Table 3).

For both groups, compared to baseline, the mean number of illness encounters per clinic for URI and ABS increased for the intervention periods from 210 to 330 and from 73 to 107 for the intervention group. For the control group the mean number of illness encounters per clinic for URI and ABS increased from 211 to 265 and from 65 to 113, respectively. The mean number of illness encounters per clinic for AOM declined in both groups from 129 to 94 and from 134 to 97, respectively.

Total antibiotic utilization was 25% for each group at baseline and, in the intervention and postintervention periods, 25% and 24%, respectively, for the intervention group, and 27% and 27%, respectively, for the control group. Intracluster correlations, estimated from the GLMMs, for URI, ABS, AOM, and BSAP% were 0.172, 0.117, 0.138, and 0.063, respectively, indicating that 17%, 12%, 14%, and 6%, respectively, of the variability in the outcomes can be explained by between-clinic differences.

## Discussion

In this cluster randomized trial among family medicine clinics, individual clinician feedback resulted in superior performance compared to clinic-level feedback for pediatric ARTIs. Specifically, individual peer comparison data yielded greater performance for URI, AOM, and ABS and a significantly greater reduction in BSAP%. Although a number of studies have demonstrated the value of individual clinician feedback, this trial was unique in directly comparing individual clinician feedback with clinic feedback.

Similar to our findings, in several pediatric patient studies where individual peer feedback was provided for ARTIs (but not compared to clinic feedback), prior studies have reported that individual clinician feedback is very effective for improving appropriate antibiotic prescribing. Gerber et al^3^ evaluated antibiotic prescribing appropriateness for ABS, streptococcal pharyngitis, and pneumonia in a hospital-affiliated network of 18 pediatric practices (162 clinicians) that received personalized, private feedback and found that BSAP% was reduced in recipients compared with controls who received no feedback.^3^ Kronman et al^4^ also showed improvement in antibiotic prescribing with a distance learning program including individual clinician feedback. In our prior work, 10 pediatric practices that received individual feedback viewable only by other clinicians in their practice significantly improved performance on URI, ABS, and AOM, reaching ≥ 90% performance for each and significant reduction in BSAP% within 18 months.^7^

As anticipated, improvements were maintained for the postintervention year because data provision continued for both groups at the clinic level, and we had shown significant improvement with this intervention alone for the control group.

Diagnostic shifting did occur with increases for both URI and ABS and declines in AOM, each occurring in both groups. Similar changes have been seen with pediatric clinics and individual peer comparison.^7^ We suspect that these changes reflect more appropriate diagnostic coding resulting from education, as suggested by others.^29,30^

Total antibiotic prescribing changed little after the intervention, as expected, since we did not focus on prescribing reduction but rather on guideline-concordant care. The reduction in use with improved URI performance likely represented a small proportion of total antibiotic use. In addition, whereas reduction in BSAP% was greater in the intervention group, it may have been even lower for both groups had we focused only on pediatric ARTIs and not included all pediatric illness encounters.

Our study had several strengths: stratified randomization of practices, continued participation and engagement of all clinics in each group during the course of the trial, and the relatively large number of geographically dispersed clinics in each trial group. Our study also had several limitations. It was conducted within a single healthcare system with a common electronic medical record and full support of an analytics group. Thus, these findings may not be broadly generalizable. Second, there was risk of contamination between groups because family medicine clinics regularly meet regionally and otherwise communicate with each other. Third, we did not estimate the extra time and resources for the intervention group, and although we received very few requests for patient data or discussion of the data provided, individual reports did not require additional time to compile each month. Fourth, we did not assess whether therapy was warranted for the diagnoses of ABS and AOM, but rather whether the chosen antibiotic was guideline-concordant. The sinusitis metric may have included patients with viral sinusitis who did not receive an antibiotic and may have penalized clinicians if no antibiotic was prescribed. We expected both groups to be affected equally, however. Finally, while the groups appeared similar at the clinic level, we did not analyze patient-level variables. We believe, however, that patient characteristics for illness encounters had little bearing on outcomes because the ARTIs studied are common in pediatric subpopulations, and our randomization approach likely provided sufficient balance in these variables between study groups.

Based on our cluster randomized trial, we conclude that individual peer comparison feedback achieves significantly greater performance improvement for 3 pediatric ARTIs and BSAP% compared with group (clinic) performance feedback. These findings add to the developing information about optimal approaches to improving appropriate antibiotic usage in the ambulatory setting. Further research is needed to understand how best to leverage peer influence to improve antibiotic prescribing, including evaluating automated clinician dashboard presentation of performance compared with direct email, and determining optimal feedback frequency.

## Supplementary material

For supplementary material accompanying this paper visit https://doi.org/10.1017/ash.2021.212. click here to view supplementary material
