# Supplementary material for: Improving antibiotic prescribing for pediatric acute respiratory tract infections: A cluster randomized trial to evaluate individual versus clinic feedback
Source: Antimicrob Steward Healthc Epidemiol. 2021 Nov 3;1(1):e43. doi: 10.1017/ash.2021.212 (PMC9495533; doi:10.1017/ash.2021.212)
Supplement: Supplementary file 1 [file ashsup.zip › S2732494X21002126sup002.docx]

Supplemental Digital Content 2, After Visit Summary

**Common Colds and antibiotics (.URIantibioticsheet as SmartPhrase for after visit summary)**

Article Body

Parents need to know that using antibiotics when they are not the right medicine will not help and may even cause harm to children.  Antibiotics are medicines used to treat [infections](http://www.healthychildren.org/English/health-issues/conditions/infections/Pages/default.aspx) and they target bacteria, not viruses. Before prescribing an antibiotic, your child's doctor will find out if it is the right medicine to treat your child's infection.

**1.  My child has a really bad cold. Why won't the doctor prescribe an antibiotic?**

Colds are caused by viruses. Antibiotics are used specifically for infections caused by bacteria. In general, most [common cold](http://www.healthychildren.org/English/health-issues/conditions/ear-nose-throat/Pages/Children-and-Colds.aspx) symptoms—such as runny nose, cough, and congestion—are mild and your child will get better without using any medicines.

**2.  Don't some colds turn into bacterial infections? So why wait to start an antibiotic?**

In most cases, bacterial infections do not follow viral infections. Using antibiotics to treat viral infections may instead lead to an infection caused by resistant bacteria. Also, your child may develop [diarrhea](http://www.healthychildren.org/English/health-issues/conditions/abdominal/Pages/Diarrhea.aspx) or other side effects. If your child develops watery diarrhea, diarrhea with blood in it, or other side effects while taking an antibiotic, call your child's doctor.

**3.  Isn't a nose draining yellow or green mucus a sign of a bacterial infection?**

During a common cold, it is normal for mucus from the nose to get thick and to change from clear to yellow or green. Symptoms often last for 10 days. 
[Sinusitis](http://www.healthychildren.org/English/health-issues/conditions/ear-nose-throat/Pages/The-Difference-Between-Sinusitis-and-a-Cold.aspx) is a term that means inflammation of the lining of the nose and sinuses. A virus or allergy can cause sinusitis and in some cases, bacteria can be the cause. 
There are certain signs that bacteria may be involved in your child's respiratory illness. If your child has a common cold with cough and green mucus that lasts longer than 10 days, or if your child has thick yellow or green mucus and a [fever](http://www.healthychildren.org/English/health-issues/conditions/fever/Pages/When-to-Call-the-Pediatrician.aspx) higher than 102°F (39°C) for at least 3 or 4 days, this may be a sign of bacterial sinusitis.

If your child has developed [bacterial sinusitis](http://pediatrics.aappublications.org/cgi/doi/10.1542/peds.2013-1071) (which is uncommon), an antibiotic may be needed. Before an antibiotic is prescribed, your child's doctor will ask about other signs and examine your child to make sure an antibiotic is the right medicine.

**4.  Do antibiotics cause any side effects?**

Side effects can occur in 1 out of every 10 children who take an antibiotic. Side effects may include rashes, [allergic reactions](http://www.healthychildren.org/English/health-issues/conditions/allergies-asthma/Pages/Allergy-Causes.aspx), nausea, diarrhea, and [stomach pain](http://www.healthychildren.org/English/health-issues/conditions/abdominal/Pages/Abdominal-Pain-in-Children.aspx). Make sure you let your child's doctor know if your child has had a reaction to antibiotics.

Sometimes a rash will occur during the time a child is taking an antibiotic. However, not all rashes are considered allergic reactions. Tell your child's doctor if you see a rash that looks like [hives](http://www.healthychildren.org/English/health-issues/conditions/skin/Pages/Hives.aspx) (red welts); this may be an allergic reaction. If your child has an allergic reaction that causes an itchy rash, or hives, this will be noted in her [medical record](http://www.healthychildren.org/English/family-life/health-management/Pages/Maintaining-a-Medical-Record.aspx).

**5.  Can antibiotics lead to resistant bacteria?**

The repeated use and misuse of antibiotics can lead to resistant bacteria. Resistant bacteria are bacteria that are no longer killed by the antibiotics commonly used to treat bacterial infection. These resistant bacteria can also be spread to other children and adults. 
**Reference: American Academy of Pediatrics www.healthychildren.org**
