# Supplementary material for: Improving antibiotic prescribing for pediatric acute respiratory tract infections: A cluster randomized trial to evaluate individual versus clinic feedback
Source: Antimicrob Steward Healthc Epidemiol. 2021 Nov 3;1(1):e43. doi: 10.1017/ash.2021.212 (PMC9495533; doi:10.1017/ash.2021.212)
Supplement: Supplementary file 1 [file ashsup.zip › S2732494X21002126sup004.docx]

Supplemental Digital Content 1, Tip Sheet

|  | |
| --- | --- |
| **URI, Acute Bacterial Sinusitis, and Acute Otitis Media in children < 18 years of age** | |
| **1.** | **Avoidance of antibiotic treatment for children with upper respiratory infections (URI)^1^**  This measure is about not treating children 3 months-18 years of age with URI with antibiotics unless they have a competing diagnosis (an antibiotic-sensitive condition such as acute otitis media, acute bacterial sinusitis, pneumonia, streptococcal pharyngitis, etc.). A higher rate means better performance. The numerator includes only patients in the age group who have not had an order for an antibiotic on or three days after the visit encounter for URI. The denominator includes all patients in the age group who had an outpatient encounter with a visit diagnosis of URI during the measurement period.  Exclusions are children who are taking antibiotics in the 30 days prior to the date of the encounter during which the visit diagnosis was established and children with a competing diagnosis (an antibiotic-sensitive condition thus requiring an antibiotic) at the time of the encounter or within three days after the diagnosis of URI. |
| **2.** | **First-line treatment for acute bacterial sinusitis (ABS) (amoxicillin preferred but amoxicillin-clavulanate acceptable as first-line treatment),^2-5^ and** |
| **3.** | **First-line treatment for acute otitis media (AOM) (amoxicillin)^6^**  These measures are about using the first-line antibiotic, based on current guidelines, for children 1-18 years of age for ABS and 6 months-12 years for AOM. The numerator is all children in the age group given first-line treatment for ABS or AOM, respectively, and the denominator is all children in the age group who had an encounter with a visit diagnosis of ABS or AOM, respectively. Exclusions are children taking any antibiotic in the 60 days prior to the date of the encounter during which the visit diagnosis was established, children  with a competing diagnosis at the time of the encounter, or in whom penicillin/amoxicillin allergy is present. |

| **How can we improve our scores?** | |
| --- | --- |
| **1.** | **Use these suggested diagnoses** and codes for URI, acute bacterial sinusitis, and acute otitis media (and set them up as favorites as below) … use the exact phrases listed when you type the phrase in the Visit Diagnosis box so we have a common language for documentation.   1. **URI (upper respiratory infection, unspecified type)** J06.9 2. **Acute bacterial sinusitis** J01.90; also save “**Acute sinusitis treated with antibiotics in the past 60 days**” (also J01.90 but the term denotes an exclusion for this measure). This phrase/code is especially helpful if a patient is given an antibiotic elsewhere (eg. another system’s ED or Urgent Care). 3. **Acute bacterial otitis media, bilateral** H66.93; **Acute bacterial otitis media, left** H66.92; **Acute bacterial otitis media, right** H66.91; **Acute bacterial otitis media, unspecified laterality** H66.90. Also save “**Otitis media treated with antibiotics in the past 60 days**” (H66.90 but the term denotes an exclusion for this measure). Phrase helpful just as with ABS.   **To set these up as favorites**: After typing the exact phrase in the Visit Diagnosis box and accepting it, right click on its displayed name. Then click ***“Add to Common Dx button list,”*** and it becomes a ***“speed button”***. Upon returning to select a Visit Diagnosis next time, you just click on the button. A wrench is present on the right of the screen and can be used to see all of your common diagnoses and to edit them as you wish. |
| **2.** | **If URI is a visit diagnosis and an antibiotic is provided**, be sure the associated visit diagnosis is an antibiotic-sensitive condition – e.g., list acute otitis media if it is treated with an antibiotic when URI is also listed as a visit diagnosis. |
| **3.** | **If a visit diagnosis has been made already and the child or adolescent patient has predominant cold symptoms** (e.g. otalgia, since ear pain, without acute otitis, was the presenting symptom), then list URI as a visit diagnosis. Note sore throat is an exclusion per the HEDIS definition. |
| How can we improve our scores? (continued) | |
| 4. | **Do not use a take/hold strategy** as this is considered in Epic as giving the patient a prescription on Day 1, even if not filled by the patient. Rather, have the parent call back if no better in 3-5 days. There is no evidence for use of the take/hold strategy with acute URIs and limited evidence for acute otitis media. |
| 5. | **Consider concerns about how parent/patient expectations for receipt of an antibiotic affect satisfaction ratings.** While 54% of providers think their patients expect antibiotics during visits for a cough or cold, only 26% of consumers report having this expectation (MMWR July 24 2015). Suggesting actions parents could take to reduce their child’s symptoms of acute URI (i.e. giving positive treatment recommendations first, like “little Johnny has a cold and here is what we can do for him…” in addition to the negative, like “your child has a virus and does not need antibiotic,” is associated with likelihood of receiving highest possible visit ratings (Mangione-Smith R, et al. Ann Fam Med 2015; 13:221-227). Patient demand is cited as a reason why pediatricians may overuse antibiotics in the ambulatory setting. In an interview study of 109 parents of children with acute URI symptoms in 4 pediatric practices, no parents said they planned to ask for antibiotics, instead expressing trust and deference to their pediatrician’s expertise (Szymczak J, Infectious Diseases Society of America annual meeting poster presentation #206, ID Week, San Diego, October 2015). |
| 6. | Finally,   1. **For URI – do not provide an antibiotic unless there is a competing visit diagnosis requiring an antibiotic.** 2. **For ABS and AOM – use first-line treatment, unless there is penicillin/amoxicillin allergy, and use the terms/codes for antibiotic use in the previous 60 days, if appropriate.** |
| *References:*   1. *Hersh AL, et al: Principles of judicious antibiotic prescribing for upper respiratory tract infections in pediatrics. Pediatrics 2013; 132:1146-1154.* 2. *Wald ER et al. Acute bacterial rhinosinusitis in children: Microbiology and treatment and Clinical features and diagnosis. In: UpToDate. Kaplan SL, Stapleton FB (Editors in Pediatrics), UpToDate, Waltham, MA (Accessed on October15, 2015).* 3. *Wald ER et al. Clinical practice guideline for the diagnosis and management of acute bacterial sinusitis in children. Pediatrics 2009; 124:9.* 4. *Chow AW et al. IDSA clinical practice guideline for acute bacterial rhinosinusitis in children and adults. Clin Infect Dis 2012; 54:e72-112.* 5. *Acute Bacterial Sinusitis in children 1-18 years of age care pathway*   [*http://iconnect.novanthealth.org/NMGPractices/NHMGQuality/CarePathways/AcuteBacteriaSinusitis/Pages/default.aspx*](about:blank)   1. *Lieberthal AS et al. The diagnosis and management of acute otitis media. Pediatrics 2013;131:e964-999.* | |
