# Supplementary material for: Improving antibiotic prescribing for pediatric acute respiratory tract infections: A cluster randomized trial to evaluate individual versus clinic feedback
Source: Antimicrob Steward Healthc Epidemiol. 2021 Nov 3;1(1):e43. doi: 10.1017/ash.2021.212 (PMC9495533; doi:10.1017/ash.2021.212)
Supplement: Supplementary file 1 [file ashsup.zip › S2732494X21002126sup005.docx]

| Supplemental Digital Content 4, Performance Feedback Example - Clinicians | | | |  |    \|  \| \| --- \| |  |  |
| --- | --- | --- | --- | --- | --- | --- | --- | --- |
|  |  |  |  |  |  |  |  |
| **Antimicrobial Stewardship: Acute Respiratory Tract Infections April 2016** |  | **Upper respiratory infection**  **no antibiotic given** | | **Acute bacterial sinusitis**  **first-line treatment** | | **Acute otitis media**  **first-line treatment** | |
|  |  | **Num/Den** | **%** | **Num/Den** | **%** | **Num/Den** | **%** |
|  |  |  |  |  |  |  |  |
| **Target** |  |  | **90%** |  | **80%** |  | **80%** |
|  |  |  |  |  |  |  |  |
| **NHMG (all pediatric and family medicine clinics)** |  | 1507/1631 | 92% | 749/891 | 84% | 887/1055 | 84% |
| **All family medicine clinics** |  | 487/569 | 86% | 220/320 | 69% | 171/215 | 80% |
|  |  |  |  |  |  |  |  |
| **Novant Health Clinic 1** |  | 10/11 | 91% | 8/24 | 33% | 5/5 | 100% |
|  |  |  |  |  |  |  |  |
| Clinician 1* |  | 2/2 | 100% | 2/6 | 33% | 1/1 | 100% |
| Clinician 2 |  | 4/4 | 100% | 4/8 | 50% | 2/2 | 100% |
| Clinician 3 |  | 1/1 | 100% | 0/2 | 0% | 0/0 | . |
| Clinician 4 |  | 3/4 | 75% | 2/8 | 25% | 2/2 | 100% |
|  |  |  |  |  |  |  |  |

*In this example, individual clinician’s names are not listed but, in monthly reports to intervention clinics, clinicians are named.
