# Supplementary material for: Improving antibiotic prescribing for pediatric acute respiratory tract infections: A cluster randomized trial to evaluate individual versus clinic feedback
Source: Antimicrob Steward Healthc Epidemiol. 2021 Nov 3;1(1):e43. doi: 10.1017/ash.2021.212 (PMC9495533; doi:10.1017/ash.2021.212)
Supplement: Supplementary file 1 [file ashsup.zip › S2732494X21002126sup003.doc]

Supplemental Digital Content 5, Measure Definitions

Appropriate Treatment for Children with Upper Respiratory Infections (URI) (NQF 0069, MU2, CMS 154)

*Definition*: Percentage of children 3 months-18 years of age who were diagnosed with upper

respiratory infection (URI) and were not dispensed an antibiotic prescription on or three days after the episode.

*Numerator*: Children without a prescription for antibiotic medication on or within three days after the outpatient visit for URI.

*Denominator*: Children 3 months to 18 years of age who had an outpatient visit with a diagnosis

of URI during the measurement period.

*Exclusions*: Children who are taking antibiotics in the 30 days prior to the date of the encounter

during which the diagnosis was established or who had an encounter with a competing diagnosis or co-morbid condition (an antibiotic-sensitive condition thus requiring an antibiotic) within three days after the initial diagnosis of URI. For 2018, encounters were excluded as well if any diagnoses other than URI were listed.

Codes: ICD9: 460, 465.0, 465.8, 465.9; ICD10: J06.9.

Appropriate Treatment for Children with Acute Bacterial Sinusitis (ABS)

*Definition*: Percentage of children 1-18 years of age with a diagnosis of acute sinusitis and

dispensed a FIRST-LINE ANTIBIOTIC (amoxicillin or amoxicillin-clavulanate).

*Numerator*: Children with the diagnosis of acute sinusitis who received either amoxicillin

or amoxicillin-clavulanate on or within three days after the outpatient visit for acute sinusitis.

*Denominator*: Children 1-18 years of age who had an outpatient visit with a diagnosis of

acute sinusitis during the measurement period

*Exclusions*: Children who are taking antibiotics in the 60 days prior to the date of the encounter

during which the diagnosis was established or who had an encounter with a competing diagnosis

or co-morbid condition as with URI. Children who have a documented allergy to penicillin

(including amoxicillin and amoxicillin-clavulanate).

Codes: ICD9: 461, 461.0, 461.1, 461.2, 461.3, 461.8, 461.9, 473.9; ICD10: J01.00, J01.10,

J01.20, J01.30, J01.40, J01.90, J32.9.

Appropriate Treatment for Children with Acute Otitis Media (AOM)

*Definition*: Percentage of children 6 months to 12 years of age with a diagnosis of AOM

and dispensed the FIRST-LINE ANTIBIOTIC (amoxicillin).

*Numerator*: Children with who received the first line antibiotic-amoxicillin on or within 3 days

after the outpatient visit for AOM.

*Denominator*: Children 6 months to 12 years of age who had an outpatient visit with a diagnosis

of acute otitis media during the measurement period

*Exclusions*: Children who are taking antibiotics in the 60 days prior to the date of the encounter

during which the diagnosis was established or who had an encounter with a competing diagnosis

or co-morbid condition as with URI. Children who have a documented allergy to penicillin

(including amoxicillin and amoxicillin-clavulanate).

Codes: ICD9: 382.00, 382.01, 382.02, 382.4, 382.9, 384.20; ICD10: H66.009, H66.019,

H66.40, H66.90, H66.91, H66.92, H66.93, H67.9, H72.90.
